# Cerebral Inefficient Activation in Schizophrenia Patients and Their Unaffected Parents during the N-Back Working Memory Task: A Family fMRI Study

**DOI:** 10.1371/journal.pone.0135468

**Published:** 2015-08-13

**Authors:** Sisi Jiang, Hao Yan, Qiang Chen, Lin Tian, Tianlan Lu, Hao-Yang Tan, Jun Yan, Dai Zhang

**Affiliations:** 1 Peking University Sixth Hospital, Beijing, 100191, China; 2 Peking University Institute of Mental Health, Beijing,100191, China; 3 Key Laboratory of Mental Health, Ministry of Health (Peking University), Beijing, 100191, China; 4 Lieber Institute for Brain Development, Baltimore, Maryland, United States of America; 5 Psychiatry and Behavioral Sciences, Johns Hopkins University School of Medicine, Baltimore, Maryland, United States of America; 6 School of Life Sciences, Tsinghua University, Beijing, 100084, China; 7 Peking University-Tsinghua University Joint Center for Life Sciences, Beijing, 100871, China; 8 PKU-IDG/McGovern Institute for Brain Research, Peking University, Beijing, China; University Of Cambridge, UNITED KINGDOM

## Abstract

**Background:**

It has been suggested that working memory deficits is a core feature of symptomatology of schizophrenia, which can be detected in patients and their unaffected relatives. The impairment of working memory has been found related to the abnormal activity of human brain regions in many functional magnetic resonance imaging (fMRI) studies. This study investigated how brain region activation was altered in schizophrenia and how it was inherited independently from performance deficits.

**Method:**

The authors used fMRI method during N-back task to assess working memory related cortical activation in four groups (N = 20 in each group, matching task performance, age, gender and education): schizophrenic patients, their unaffected biological parents, young healthy controls for the patients and older healthy controls for their parents.

**Results:**

Compared to healthy controls, patients showed an exaggerated response in the right dorsolateral prefrontal cortex (brodmann area [BA] 46) and bilateral ventrolateral prefrontal cortex, and had reduced activation in bilateral dorsolateral prefrontal cortex (BA 9). In the conjunction analysis, the effect of genetic risk (parents versus older control) shared significantly overlapped activation with effect of disease (patients versus young control) in the right middle frontal gyrus (BA 46) and left inferior parietal gyrus (BA 40).

**Conclusions:**

Physiological inefficiency of dorsal prefrontal cortex and compensation involvement of ventral prefrontal cortex in working memory function may one physiological characteristics of schizophrenia. And relatively inefficient activation in dorsolateral prefrontal cortex probably can be a promising intermediate phenotype for schizophrenia.

## Introduction

Schizophrenia is a highly heritable disorder with estimated heritability of approximately 81% [1,2]. As a key feature of the symptomatology of schizophrenia, cognitive impairment has been reported in many domains, including working memory, executive function, attention, language and memory [3,4]. Evidence from meta-analyses and studies of unaffected relatives of schizophrenic patients suggests that cognitive impairment is familial and related to the genetic vulnerability of schizophrenia [5,6]. The impairment can be detected in attenuated form in individuals at high risk for schizophrenia who are unaffected and have never been exposed to treatment [7]. Therefore, the cognitive dysfunction could be an inherent biological phenomenon. Studying the cognitive impairment and related neural substrate in patients with schizophrenia and their unaffected first-degree relatives may be an effective approach to understanding the pathology of schizophrenia and underlying genetic mechanism.

Working memory is considered to be a core cognitive domain impaired in patients with schizophrenia [8]. As a crucial part of higher cognitive functions, working memory enables us to temporarily hold and manipulate information with a limited capacity [9]. According to Baddeley's model, working memory contains four major components: the central executive, visuo-spatial sketch pad, phonological loop, and episodic buffer. Central executive supports the manipulation and transformation of information held within the storage buffers; and visuo-spatial sketch pad /phonological loop is a short-term storage buffer for visual/verbal information [9]. Previous studies have shown that, the central executive is associated with the function of dorsolateral prefrontal cortex (DLPFC), while the storage buffers is associated with both inferior frontal, including ventrolateral prefrontal cortex (VLPFC), and posterior parietal function [10,11]. N-back task is a canonical working memory task requiring on-line monitoring, updating, and manipulating of information [12]. Twins study using functional magnetic resonance imaging (fMRI) has suggested that patterns of brain activity related to N-back task were heritable, with the high estimate value (40–65%) in regions of the working memory related brain network, such as the inferior, middle, and superior frontal gyri [13].

Converging evidences suggest that working memory dysfunction in patients with schizophrenia may be due to deficits in dorsolateral prefrontal cortex (DLPFC) [14,15,16]. Disruptions in working memory and associated neural activities have been found not only in schizophrenic patients but also in their first-degree relatives [17]. Deficits in cortical information processing have been known as an attractive intermediate phenotypes related to schizophrenia, especially the inefficient activation of DLPFC in working memory task. For example, Callicott et al. examined N-back task related fMRI activity in unaffected siblings of patients with schizophrenia. In this study, exaggerated response in the right DLPFC were found in the siblings and same exaggerated response was verified in a planned replication [7].

Studies investigating the heritability of working memory for schizophrenia mostly examined brain activation alternation in siblings or offspring of schizophrenic patients [7,18,19,20,21]. However, the parents of patients were rarely studied so far and it is unknown if potentially heritable aspects of brain dysfunction may also be present in the unaffected parents who are already beyond the age of risk for schizophrenia. The current study therefore investigated the working memory related brain activity in schizophrenic patients and their unaffected parents by using N-back fMRI data in a voxel-wise whole brain analysis. We hypothesized that unaffected parents of patients with schizophrenia would still manifest altered prefrontal activation.

## Materials and Methods

### Ethics Statement

The research was approved by the Medical Research Ethics Committee of the Institute of Mental Health, Peking University. All participants were given detailed information regarding the purpose and procedures of the study. Only the patients who had capacity to consent would be invited to participate in the study. All participants enrolled in this study signed the written consents by themselves.

### Subjects

In total, 155 Chinese Han subjects were enrolled in this study, including 33 schizophrenic patients (SCZ), 63 their unaffected biological parents (PAT) (31 fathers, 32 mothers), 30 young healthy controls for the SCZ (NCS) and 29 old healthy controls for the PAT (NCP).

The SCZ and PAT groups were recruited from the Institute of Mental Health, Peking University. Patients with schizophrenia all met the ICD-10 diagnostic criteria for paranoid schizophrenia, which was ensured by two psychiatrists. All patients were receiving antipsychotic medications when scanning, and the dosages were converted to the equivalent dose for chlorpromazine [22]. The severity of disease was evaluated by an experienced psychiatrist using the Positive and Negative Syndrome Scale (PANSS). Patients treated with electroconvulsive therapy within 6 months or with a history of serious medical illness were excluded from the current study. The NCS and NCP groups were recruited from the local communities and well matched with patients and parents of patients, respectively, for age, sex and education levels. None of healthy controls reported having first- or second-degree relatives with schizophrenia spectrum disorders. All participants were assessed to be right-handed using the Edinburgh Handedness Inventory [23] and had no intracranial pathology, history of brain injury, neurological disorder, or alcohol/substance abuse. All first-degree relatives and normal controls had no personal history of psychiatric illness confirmed by an unstructured interview. Performances of executive control with Wisconsin Card Sorting Test (WCST) and episodic memory (EM) with subtest of Logical Memory (LM) from the Wechsler Memory Scale-Chinese Revised (WMS-CR) were assessed in all participants.

### Neuroimaging

#### N-back Task

All subjects underwent two sessions of functional magnetic resonance imaging (fMRI) scanning during an N-back working memory task which has been described in detail elsewhere [24]. Stimuli were presented on a rear projection screen placed at the subject’s feet. Subjects saw the screen from a mirror attached to the head coil. Responses were recorded via a fiber-optic response box with buttons arrayed in the same configuration as the stimuli presented on the screen. Four numbers 1, 2, 3, and 4 were displayed at four points of a diamond-shaped box one at a time in a pseudo-randomized order at the rate of 0.5 Hz (every 2 s); each stimulus appeared 500 ms and followed by a 1500 ms blank diamond-shaped box (the response period). This block designed N-back task included eight blocks in each session and one block consisted of 3 conditions (20 s for each condition). 0-back and 2-back conditions were alternated with rest condition during which subjects were asked to relax with their eyes open. Subjects were asked to press the button according to position of the current number they saw in the 0-back condition, and to press the button according to position of the previous number but one in the 2-back condition [25].

Immediately before scanning, all subjects were given a training session to ensure that they well understood the task design and knew how to perform with the response box. The training stopped until they achieved 90% of the targets in the 0-back condition.

Performance was recorded as percentage of correct response (accuracy) for 0-back and 2-back condition respectively in each block. The block with 2-back accuracy above 62.5% was set as a good block. In order to make sure the subjects concentrated and took their effort to complete the task, only sessions having 2 good blocks or more were included in the subsequent fMRI data analysis. During quality control(QC) of task performance, both two sessions were discarded in 30 subjects (8 in SCZ, 3 in NCS, 16 in PAT, 3 in NCP) and one session was removed in 16 subjects (3 in SCZ, 2 in NCS, 9 in PAT, 2 in NCP).

#### Image Acquisition

MRI scanning was conducted using a Magnetom Trio (Siemens Medical System, Erlangen, Germany) 3T system running at the Peking University third hospital. Foam pads were used to reduce head motion and scanner noise. Three-dimensional T1-weighted images were acquired in a sagittal orientation employing a 3D-MPRAGE sequence with the following parameters: time repetition (TR) = 2350 ms, time echo (TE) = 3.44 ms, flip angle = 7°, matrix size = 256 × 256, field of view (FOV) = 256 × 256 mm^2^, 192 sagittal slices, slice thickness = 1 mm, acquisition voxel size = 1.0 × 1.0 × 1.5 mm^3^, total acquisition time = 363 s. Two 8.8 min gradient echo planar images were acquired (TR = 2 s, TE = 30 ms, flip angle = 90°, matrix size = 64 × 64, FOV = 220 × 220 mm², thickness/slice gap = 4/0.8 mm, voxel size = 3.438 × 3.438 × 4.8 m³). Each fMRI session consisted of 267 images. There are 3 dummy scans before each run. A three-dimensional reconstruction of T1 images was carried out to screen for severe artifacts and anomalies of brain. 2 subjects in PAT group were excluded because of old cerebral Infarction.

#### fMRI image preprocessing

fMRI image preprocessing was implemented using SPM8 (http://www.fil.ion.ucl.ac.uk/spm) following standard procedures. All images were realigned to the first scan of first session. Structural image of each subject was co-registered to the corresponding mean of the realigned image, and then was segmented into gray matter and white matter using SPM8 Eastern Asian template. The non-linear transformation matrix was used in normalization to write out the normalized images. All images were resliced (2 × 2 × 2 m³). Low frequency components were removed using a low-pass filter (128 s) and images were spatially smoothed using a Gaussian filter (8 mm full-width half maximum; FWHM). An autoregressive AR (1) model was used to account for serial correlation.

In this step, QC was carried out by checking head motion and normalization. Head motion was detected following a procedure from ArtRepair toolbox. To include the useful data as much as possible, we kept the fMRI sessions with sudden and transient head motion within the acceptable criteria (2–3 mm in translation or2-3°in rotation). Blocks with head motion within above criteria were marked as bad blocks and were set as an error “condition” in first level analysis. Only sessions with severe head motions (≥3 mm in translation or ≥ 3° in rotation) were removed from the subsequent analysis. Both sessions were excluded in 7 subjects (3 in SCZ, 3 in PAT and 1 in NCP) and one session was removed in 8 subjects (3 in SCZ, 1 in NCS, 3 in PAT and 1 in NCP). No severe distortion was found in normalization.

#### Statistical analysis

It has been shown that the cerebral cortex activation is sensitive to the behavioral performance differences [26,27,28], so we further matched task performance between four groups based on block-wise averaged 2-back accuracy. This selected 20 well matched subjects with high quality MRI data in each group (80 subjects in total) for our study.

At an individual level, relative changes in regional Blood Oxygenation Level Dependent (BOLD) signal were assessed using a GLM which specified the onset and duration of the two n-back conditions, rest conditions and instruction periods. Blocks with 2-back accuracy lower than 62.5% or head motion greater than 2–3 mm translation or rotation greater than 2–3 degree were marked as bad blocks and were set as error here. Movement parameters were entered as nuisance covariates. A canonical hemodynamic response function (HRF) was used to model the fMRI responses. Working memory related processing was defined as contrast between 2-back condition versus 0-back condition for each subject [18]. The numbers of good blocks between different subjects were diverse. To remain as many samples as possible, statistical contrast images of the 2 back versus 0 back condition for each good block were carried to second-level analysis[29].

In second level analysis, a repeated measure ANOVA was conducted using flexible factorial design to account for variance of number of good blocks for each subject as a repeated factor, and “group” as the between-subject factor (four levels: SCZ, NCS, PAT, NCP). From this model we created second-level contrasts of interest. Firstly, the working memory related activation of each group was contrast under the flexible factorial design. In the current study, we limited our analysis to the positive activation in each group. To explore the between group difference of the working memory activation, a common activation mask was generated by interacting the common brain region where all four groups have significant positive activation.

To identify differences between SCZ and NCS, PAT and NCP, respectively, the two-sample t-tests were used, as post hoc analysis under flexible factorial design, during which, the mask described above was used. Compared to matched healthy controls, brain activation of schizophrenic patients may be different affected by ‘state associated with disease’ and ‘genetic load’. And activation of parents of schizophrenia are various from their matched controls not only affected by ‘genetic load’ but also possible by ‘long-term life experience and stress of special role’. To further examine the effect of the familial risk of schizophrenia, therefore, we carried out a conjunction contrast as SCZ>NCS conjunction with PAT>NCP within the framework of SPM [30]. It is defined that conjunction of two statements is true if and only if both of them are true [31,32]. In our study, the conjunction activation was associated with ‘genetic load’. Our conjunction contrast thus focused on the brain regions with higher (inefficient) activation associated with schizophrenia and potential familial risk for schizophrenia in the unaffected parents of patients versus corresponding healthy controls. Statistical significance was set at *p* < 0.05 using family-wise error (FWE) correction in all tests during group analysis. We calculated effect size (ES) by cohen’d values of each peak point of conjunction analysis by a web-based tool, Effect Size Calculators (http://www.uccs.edu/lbecker/index.html).

## Results

### Demographic and Behavioral Data

80 subjects (20 for each group) were included in the final analysis. All four groups were well matched for gender and years of education. Age at scan were well matched between SCZ and NCS, as well as PAT and NCP. The four groups did not show significant differences in the number of good blocks, 2-back accuracy and performance of WCST. One patient did not finish the LM assessment. Performance of LM was found to be significant different (*p* = 0.045) between patients and their control. The details of demographical and clinical characters are shown in Table 1.

**Table 1:** Demographic, clinical and cognitive details for each group.

Variable	SCZ	NCS	*P*	PAT	NCP	*P*
	(n = 20)	(n = 20)		(n = 20)	(n = 20)	
**Gender(male/female)**						0.8^a^
**Male (n(%))**	13	16		11	10	
**Female (n(%))**	7	11		9	10	
**Age at scan(y)**	22.7(3.8)	23.1(3.1)	0.7^b^	50.7(5.0)	51.8(5.9)	0.5^b^
**Educational level(y)**	13.8(1.8)	14.2(1.6)		13.5(3.2)	13.8(2.6)	0.9^c^
**Number of good blocks**	10.40(3.87)	12.40(4.19)		11.05(4.31)	10.35(4.06)	0.36^c^
**2back accuracy**	0.86(0.09)	0.88(0.09)		0.83(0.10)	0.84(0.96)	0.33^c^
**Age of disease onset(y)**	19.0(2.6)					
**Illness duration(m)**	40.6(36.9)					
**PANSS sum score**	66.5(10.9)					
**PANSS positive score**	18.6(4.7)					
**PANSS negative score**	16.0(4.5)					
**PANSS general score**	31.9(4.7)					
**CPZ-eq at scan(mg/d)**	402.0(233.4)					
**WCST category score**	3.9(2.3)	4.8(2.0)	0.2 ^b^	3.2(2.4)	2.9(2.3)	0.7 ^b^
**LM**	9.4(2.3)	10.9(2.0)	0.045 ^b^	8.3(2.4)	9.2(1.7)	0.2 ^b^

Significance threshold defined at *p*<0.05

P^a^ p value for Pearson’s χ^2^-test

*P* ^b^ p value for Two-Sample *t* Test

*P* ^c^ p value for one way ANOVA

CPZ-eq, chlorpromazine equivalents.

### *fMRI* results

#### Task-related activation

In each group, significant activation (p<0.05, FWE corrected) were observed in middle frontal gyrus, supplementary motor area, inferior parietal lobule, inferior temporal gyrus and cerebellum (See S1 Fig and S1 Table in detail).

#### Disease-related activation

**Fig 1:**
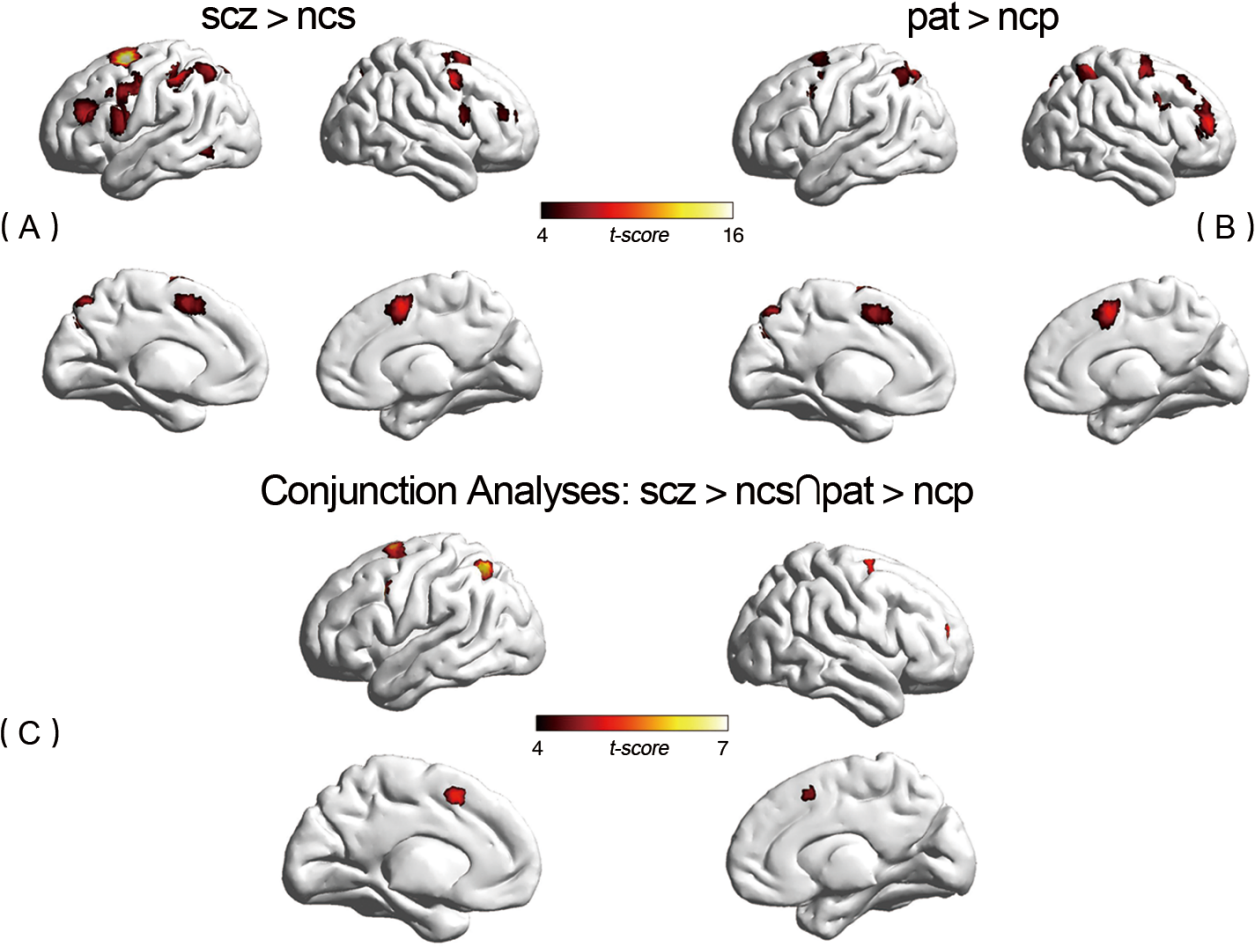
Discrepant Activation between Groups and in Conjunction Analysis Using the BrainNet Viewer software (http://www.nitrc.org/projects/bnv/) [33]. (A) Between-group hyper activation regions related to disease (SCZ > NCS). Regions with significantly (*p*<0.05, FWE corrected) activation. (B) Between-group hyper activation regions related to familial risk (PAT > NCP). Regions with significantly (p<0.05, FWE corrected) activation. (C) Conjunction analyses depict significant overlap in activity related to disease and activity related to familial risk (SCZ > NCS∩PAT > NCP) (*p*<0.05, FWE corrected). Clusters are depicted in the right middle frontal gyrus, bilateral precentral gyrus, left inferior parietal gyrus, bilateral superior frontal gyrus, and left supplementary motor area.

**Fig 2:**
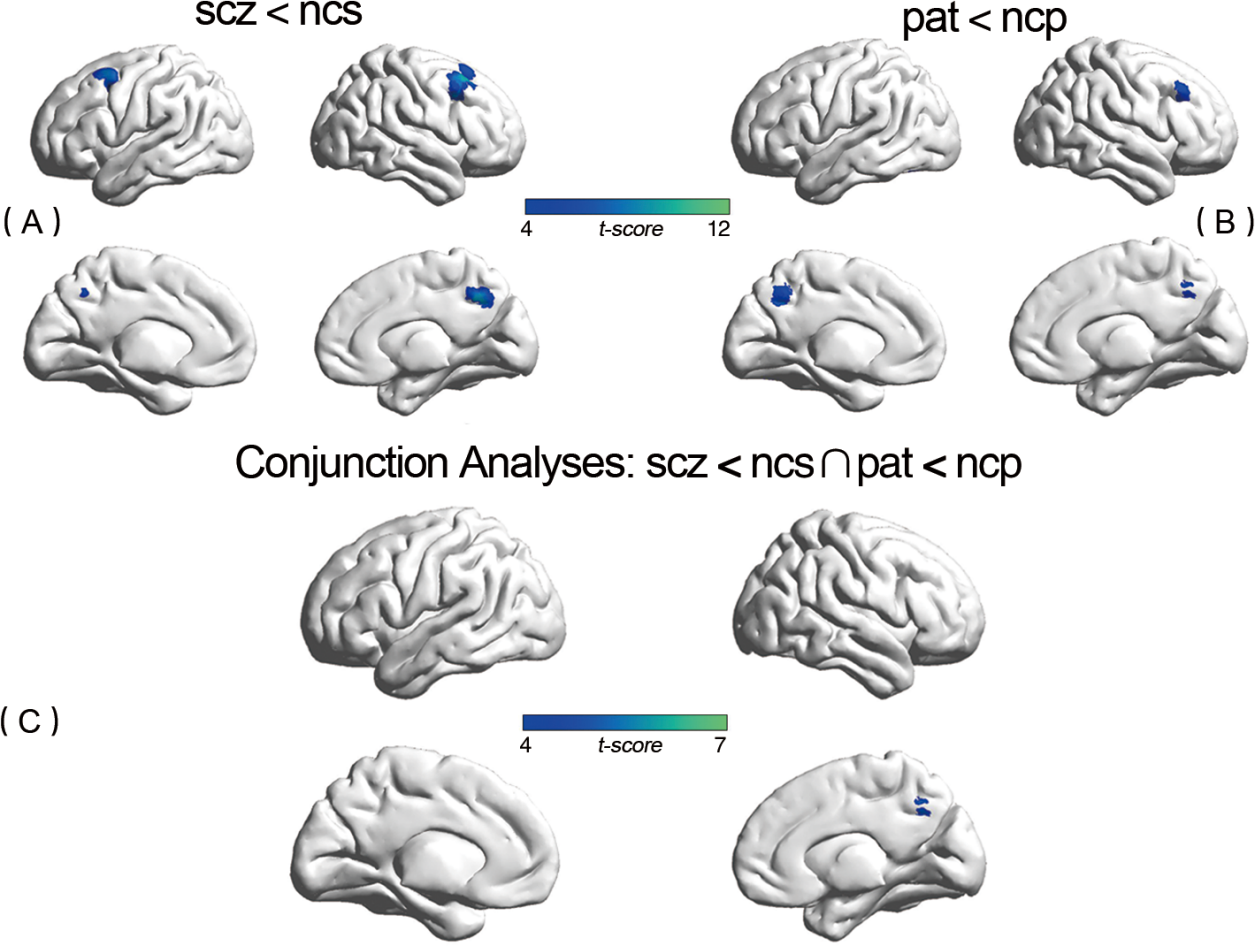
Discrepant Activation between Groups and in Conjunction Analysis Using the BrainNet Viewer software (^http://www.nitrc.org/projects/bnv/^) [33]. (A) Between-group reduced activation regions related to disease (SCZ < NCS). Regions with significantly (*p*<0.05, FWE corrected) activation. (B) Between-group reduced activation regions related to familial risk (PAT < NCP). Regions with significantly (p<0.05, FWE corrected) activation. (C) Conjunction analyses depict significant overlap in activity related to disease and activity related to familial risk (SCZ < NCS∩PAT < NCP) (*p*<0.05, FWE corrected). Clusters are depicted in the right precuneus gyrus.

Compared with controls, patients showed significantly greater activation (p<0.05, FWE corrected) in several areas related to working memory, including bilateral frontal gyrus (Brodmann area [BA] 6/40/44/45/46), bilateral parietal lobule (BA 7/40) and right Insula (BA 48). In particular, we found increased activation in right dorsolateral prefrontal cortex (DLPFC) (BA 46) and bilateral ventrolateral prefrontal cortex (VLPFC) (BA44/45) in patients compared to controls. However, compared to normal controls, patients showed significantly reduced activation in bilateral DLPFC (BA 9). (see Figs 1A and 2A and S2 Table in detail).

#### Familial risk-related activation

Compared with age-matched healthy controls, parents of patients showed significantly greater task-elicited activation (p<0.05, FWE corrected) in bilateral frontal gyrus (BA 6/9/44/45/46/48), bilateral parietal gyrus (BA 7/40), and left supplementary motor area (BA 32). The regions showed significantly reduced activation were the bilateral middle frontal gyrus (BA45/46) and precuneus gyrus. In the frontal lobe, unaffected parents showed increased activation in bilateral DLPFC (BA 9/46) and right VLPFC (BA 44/45) compared to controls, while having reduced activation in bilateral VLPFC (BA 45/47). (See Figs 1B and 2B and S3 Table in detail)

We used conjunction analyses to further investigate the familial risk function of impaired cerebral activation in schizophrenia. In our study, the effect of genetic risk (PAT>NCP) shared significantly (p<0.05,FWE corrected) overlapped activation with effect of disease (SCZ>NCS) in the right middle frontal gyrus (BA 46), bilateral precentral gyrus (BA 6), left inferior parietal gyrus (BA 40), bilateral superior frontal gyrus (BA 6),and left supplementary motor area (BA 32). In addition, the effect of genetic risk (PAT>NCP) shared significantly (p<0.05, FWE corrected) overlap with effect of disease (SCZ>NCS) in the right precuneus gyrus. Most of discrepant regions in conjunction analysis shew a medium effect size. (See Figs 1C and 2C and Table 2 in detail)

**Table 2:** Conjunction analyses show significant overlap in different activity between SCZ>NCS ∩PPT>NCP and SCZ<NCS ∩PPT<NCP (*P*<0.05, FWE).

Brain region	BA	Left(L)						Right(R)					
		MNI (in mm)			size	t-value	ES	MNI (in mm)			size	t-value	ES
		x	y	z				x	y	z			
**SCZ>NCS and PPT>NCP**													
Precentral Gyrus	6	-48	6	38	56	6.38	0.44						
Inferior Parietal Lobule	40	-38	-56	52	56	6.25	0.43						
Superior Frontal Gyrus	6	-20	2	66	59	5.99	0.41						
Superior Frontal Gyrus	6							30	-4	62	20	5.57	0.38
Supplementary Motor Gyrus	32	-4	12	52	27	5.28	0.36						
Middle Frontal Gyrus	46							36	52	18	13	5.23	0.36
Precentral Gyrus	6							52	6	36	13	5.09	0.35
**SCZ**<**NCS and PPT**<**NCP**													
Precuneus Gyrus								10	-58	42	23	5.96	0.41

## Discussion

There is to our knowledge no other study on potential cognitive and genetic effects associated with schizophrenia using patients and their unaffected parents. In our current study, we investigated cerebral activity during the N-back working memory fMRI task in schizophrenia families and found the shared inefficient activation in patients with schizophrenia and their unaffected parents.

Although each group activated regions of “fronto-parietal-cerebellar” circuit in the working memory network that was found in a number of previous studies using the same task [34,35], we also found significant between-group differences in the activation level of the above regions suggesting that even parents of schizophrenia patients beyond the age of risk of illness manifest relative prefrontal cortical dysfunction.

Specifically, we found that schizophrenia patients showed increased activation in DLPFC and VLPFC, although some previous studies found central executive deficits only in DLPFC [11,36]. In our study, we carefully controlled for task accuracy before statistical analysis. Therefore, our findings suggest that a greater extent of neural activity was engaged in patients to achieve the same working memory performance, consistent with patients being more inefficient. In addition, the increased activation in DLPFC and VLPFC provided evidence for the hypothesis that patients required not only more activation in DLPFC to drive working memory performance but perhaps also compensatory activation from VLPFC, a region engaged in simpler rehearsal operation [15,37]. Previous studies suggest that DLPFC was selectively involved in successfully processing working memory executive load in healthy individuals [7]. Our data is consistent with patients requiring greater inefficient activation of DLPFC to perform at the same level, and that patients may have difficulty with processes attributed to higher-order executive process of working memory [11,38,39]. VLPFC has been suggested to involve in less complex process such as rehearsal in healthy controls, but possibly serve as a compensation to dysfunctional DLPFC in schizophrenia. This “inefficient” and “compensation” interpretation has been supported by correlation studies and functional connectivity studies. For example, Tan et al. reported that working memory performance was correlated with DLPFC activation in healthy individuals but correlated more with VLPFC in patients; and posterior parietal cortex had relatively greater functional connectivity with the DLPFC in healthy individuals while increased connectivity with VLPFC in schizophrenia patients [14].

Another finding was the opposing activation patterns within the DLPFC (i.e. BAs 9 and 46) in patients. Compared with healthy controls, patients with schizophrenia had higher activation in BA 46 but lower activation in BA 9. The findings suggest functional differentiation of DLPFC and suggest a possibility that BA 9 and BA46 may be differentially affected in our schizophrenia patients. The anterior and posterior functional heterogeneity of DLPFC had been found in another co-activation-based parcellation fMRI study [40]. In their study, they suggested the anterior DLPFC network to be more strongly associated with attention and action inhibition processes, whereas the posterior DLPFC network was more strongly related to action execution and working memory. In addition, a recent study demonstrated that the alterations of genetic expression pattern and cell density between schizophrenic patients and normal controls were limited to BA 9, which also supports regional specificity of DLPFC abnormalities in schizophrenia [41].

Besides the illness effect, the genetic risk effect of the working memory impairment was another important aspect in the current study. As we all known, there is a lower lifetime risk of developing schizophrenia in parents (6%), compared with sibling (9%) or offspring (13%)[42], and parents have already passed the age of peak risk for schizophrenia and are unlikely to have further onset of schizophrenia. Therefore, including parents of patients allow us to explore the genetic risk effect of disease independent of onset of schizophrenia. In our study, the conjunction analyses suggest that the brain activation of DLPFC (BA 46) was significantly increased in both patients and unaffected parent groups. Because parents are unaffected by schizophrenia, we may attribute the findings to their having some genetic risk for schizophrenia. The higher DLPFC activation in unaffected parents is consistent with previous sibling studies [7,43,44] and suggest new evidence to support the inefficient DLPFC activity in working memory processes that persists into later periods of adulthood and maybe one intermediate phenotype of schizophrenia. Intermediate phenotype is phenotype which intermediate between the cellular effects of susceptibility genes and the manifest psychopathology, which may developed to be tools for gene discovery, improving the power of association studies by reducing phenotypic heterogeneity[45,46].Recent years, some genetic and molecular studies had already tried to explore the possible pathophysiology of schizophrenia based on the view that inefficient activation of DLPFC in schizophrenia is heritable and replicable [7,47,48]. For example, in one of these studies, Egan et al found that Val allele of COMT, one of the most robust susceptibility gene for schizophrenia, increasing dopamine catabolism in prefrontal lobe, impairs prefrontal cognitive function, and lightly increases risk for schizophrenia [49]. In addition, the inferior parietal lobule was another region showed increased activation in both patients and their unaffected parents. It is consistent with some recent studies which found hyperactivation of inferior parietal lobule in unaffected siblings of schizophrenic patients in working memory task [50,51]. Therefore, IPL could also be associated with the genetic susceptibility of schizophrenia.

However, we did not detect the VLPFC effects during the conjunction analysis between schizophrenia patients and their parents. One possible explanation for the negative finding in VLPFC was that the deficits in VLPFC observed in schizophrenia patients are unlikely related to genetic risk for schizophrenia but more likely related to factors such as compensation[19].

## Issues

Deficits in other cognitive domains except working memory may affect brain activation as well. As shown in previous review, impairment of working memory, executive control, and episodic memory had been consistently found in schizophrenia researches[19]. In our study, performance of executive control (WCST) were not significant difference between groups. However, performance of episodic memory (LM) was found to be different (*p* = 0.045) between patients and their control (see S4 Table For detail). Though this difference cannot survive the correction for multiple comparisons, we still assessed whether our all findings of conjunction analysis are due to deficits in episodic memory. A pearson correlation analysis was conducted between the mean activation level of the regions in the conjunction analysis and performance of LM. A correlation (p = 0.031, uncorrected) was found only with the shared hypoactivation of the Precuneus (‘SCZ<NCS and PPT<NCP’). However, it obviously won’t survive the correction for multiple comparisons either. In short, we may infer that our main findings were not likely to be induced by the deficits in another (non-WM) cognitive domain.

There are two possible confounding factors in our findings. First, all patients were using antipsychotics during fMRI scanning, and medication usage may affect their functional brain activation. However, the Pearson correlation analysis was carried out between the mean activation level of the regions in the conjunction analysis and chlorpromazine equivalents in patients and no significant correlation (p<0.05) were found. We may infer that medication usage has no significant influence on our results. Second, we cannot exclude the potential effect of special life-experiences, emotional state and stress of special role in unaffected parents groups, such as stress or stigma, living with a sick child. There is necessary to include assessments of life experiences and emotional state for parents of patients in the future work.

## Conclusion

This study provides evidence of physiological inefficiency of dorsal prefrontal cortex and compensation involvement of ventral prefrontal cortex in working memory function in performance-matched schizophrenic patients relative to healthy controls. Data from the unaffected parents of schizophrenic patients suggest that the inefficient activation of dorsal prefrontal cortex and inferior parietal lobule could be genetic trait markers for schizophrenia that persists beyond mid-adulthood.

## Supporting Information

S1 Fig Whole Cerebral Regions Positive Related to the Working Memory N-back Task (*p*<0.05, FWE corrected) for Each Group. Using the BrainNet Viewer Software (http://www.nitrc.org/projects/bnv/) [33].
(TIF)

S1 Table Brain areas showing significant task related positive activation (2back>0back) in each group (p<0.05, FWE corrected, with a minimum cluster size of 20 voxels). (DOCX)

S2 Table Significant differences in brain activation during n-back task (2back>0back) between patients of schizophrenia and young healthy controls. Using a common activation mask generated by interacting common brain region where all four groups have significant positive activation (*P*<0.05, FWE; with a minimum cluster size of 20 voxels).
(DOCX)

S3 Table Significant differences in brain activation during n-back task (2back>0back) between unaffected parents of patients and old healthy controls. Using a common activation mask generated by interacting common brain region where all four groups have significant positive activation (*P*<0.05, FWE; with a minimum cluster size of 20 voxels).
(DOCX)

S4 Table Cognitive (WCST and LM) performance in four groups: the patients, young healthy controls, parents and older unaffected controls. (DOCX)
