# Supplementary figures and images for: Cerebral Inefficient Activation in Schizophrenia Patients and Their Unaffected Parents during the N-Back Working Memory Task: A Family fMRI Study
Source: PLoS One. 2015 Aug 13;10(8):e0135468. doi: 10.1371/journal.pone.0135468 (PMC4536207; doi:10.1371/journal.pone.0135468)

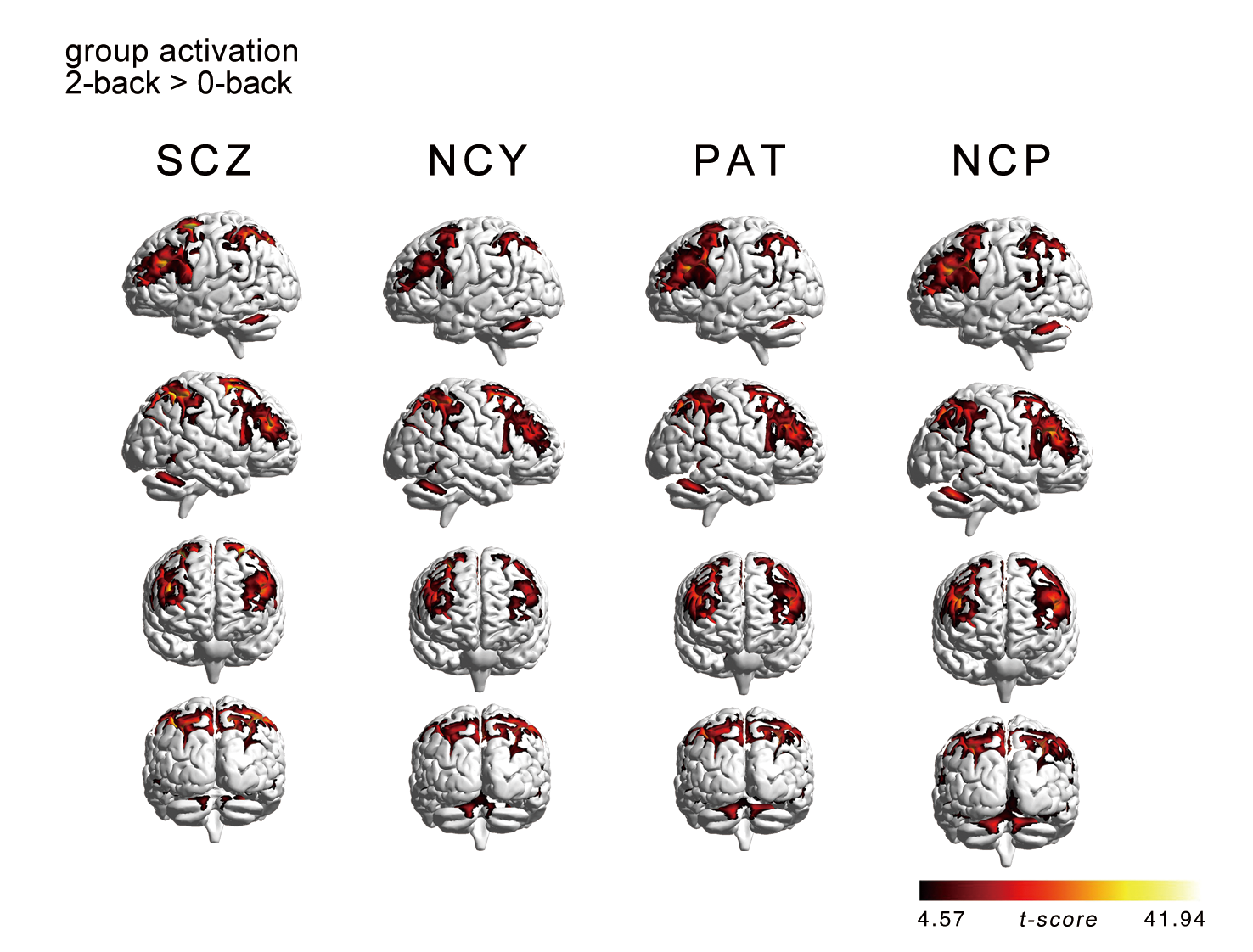

Supplement: S1 Fig — Using the BrainNet Viewer Software (http://www.nitrc.org/projects/bnv/) [33]. (TIF) [file pone.0135468.s001.tif]
