# Supplementary material for: Cerebral Inefficient Activation in Schizophrenia Patients and Their Unaffected Parents during the N-Back Working Memory Task: A Family fMRI Study
Source: PLoS One. 2015 Aug 13;10(8):e0135468. doi: 10.1371/journal.pone.0135468 (PMC4536207; doi:10.1371/journal.pone.0135468)
Supplement: S1 Table — (DOCX) [file pone.0135468.s002.docx]

**Table S1.** Brain areas showing significant task related positive activation (2back>0back) (*p*<0.05; family-wise error correction; *P*<0.05, FWE,

with a minimum cluster size of 20 voxels) in four groups.

| Brain region | BA(L/R) | Left(L) | | |  |  | Right(R) | | |  |  |
| --- | --- | --- | --- | --- | --- | --- | --- | --- | --- | --- | --- |
|  |  | MNI coordinaes (in mm) | | | size | t-value  (voxels) | MNI coordinaes (in mm) | | | size | t-value  (voxels) |
|  |  | x | y | z |  |  | x | y | z |  |  |
| ***Schizophrenia patients*** |  |  |  |  |  |  |  |  |  |  |  |
| Middle Frontal Gyrus | 6 | -28 | 4 | 60 | 27094 | 41.94 |  |  |  |  |  |
| Superior Frontal Gyrus | 8 |  |  |  |  |  | 26 | 8 | 60 |  | 41.61 |
| Supplementary Motor Area | 32 | -4 | 14 | 50 |  | 35.06 |  |  |  |  |  |
| Inferior Parietal Lobule | 40 |  |  |  |  |  | 38 | -46 | 42 | 19844 | 36.84 |
| SupraMarginal Gyrus | 40 |  |  |  |  |  | 44 | -38 | 40 |  | 36.81 |
| Precuneus Gyrus | 7 | -10 | -66 | 48 |  | 36.48 |  |  |  |  |  |
| Cerebellum |  |  |  |  |  |  | 32 | -62 | -28 | 1650 | 18.86 |
| Inferior Temporal Gyrus | 37 |  |  |  |  |  | 54 | -52 | -10 |  | 11.37 |
| Cerebellum |  |  |  |  |  |  | 10 | -74 | -26 |  | 10.38 |
| Cerebellar Vermis |  |  |  |  |  |  | 2 | -46 | -18 | 321 | 6.79 |
| ***Young healthy controls*** |  |  |  |  |  |  |  |  |  |  |  |
| Middle Frontal Gyrus | 8 |  |  |  |  |  | 30 | 10 | 54 | 23930 | 34.21 |
| Middle Frontal Gyrus | 8 | -28 | 8 | 54 |  | 27.15 |  |  |  |  |  |
| Supplementary Motor Area | 6 |  |  |  |  |  | 6 | 20 | 46 |  | 26.02 |
| Inferior Parietal Lobule | 40 |  |  |  |  |  | 38 | -52 | 50 | 18422 | 30.86 |
| Precuneus | 7 |  |  |  |  |  | 10 | -64 | 52 |  | 30.67 |
| Superior Parietal Lobule | 7 | -28 | -64 | 42 |  | 26.22 |  |  |  |  |  |
| Cerebellum |  | -34 | -60 | -30 | 4502 | 18.46 |  |  |  |  |  |
| Cerebellum |  |  |  |  |  |  | 32 | -60 | -30 |  | 18.06 |
| Cerebellum |  | -10 | -74 | -28 |  | 17.61 |  |  |  |  |  |
| ***Parents of patients*** |  |  |  |  |  |  |  |  |  |  |  |
| Supplementary Motor Area | 32 | 0 | 20 | 48 | 33241 | 30.80 |  |  |  |  |  |
| Inferior Frontal Gyrus | 45 | -42 | 28 | 28 |  | 30.67 |  |  |  |  |  |
| Precentral Gyrus | 44 | -46 | 8 | 36 |  | 28.99 |  |  |  |  |  |
| Inferior Parietal Lobule | 40 | -44 | -48 | 44 | 18698 | 27.07 |  |  |  |  |  |
| Inferior Parietal Lobule | 40 | -30 | -52 | 40 |  | 26.92 |  |  |  |  |  |
| Inferior Parietal Lobule | 7 | -36 | -58 | 50 |  | 26.80 |  |  |  |  |  |
| Cerebellum |  |  |  |  |  |  | 34 | -62 | -30 | 6861 | 20.87 |
| Cerebellum |  | -32 | -64 | -28 |  | 19.94 |  |  |  |  |  |
| Cerebellum |  | -10 | -76 | -28 |  | 18.71 |  |  |  |  |  |
| Middle Frontal Gyrus |  |  |  |  |  |  | 40 | 46 | -8 | 24 | 11.02 |
| ***Old healthy controls*** |  |  |  |  |  |  |  |  |  |  |  |
| Inferior Parietal Lobule | 7 | -30 | -62 | 42 | 69053 | 30.52 |  |  |  |  |  |
| Inferior Parietal Lobule | 40 | -42 | -44 | 38 |  | 29.82 |  |  |  |  |  |
| Inferior Parietal Lobule | 40 |  |  |  |  |  | 44 | -44 | 42 |  | 29.48 |
| Middle Frontal Gyrus | 47 |  |  |  |  |  | 38 | 50 | -8 | 24 | 12.74 |
