# Supplementary material for: Cerebral Inefficient Activation in Schizophrenia Patients and Their Unaffected Parents during the N-Back Working Memory Task: A Family fMRI Study
Source: PLoS One. 2015 Aug 13;10(8):e0135468. doi: 10.1371/journal.pone.0135468 (PMC4536207; doi:10.1371/journal.pone.0135468)
Supplement: S2 Table — Using a common activation mask generated by interacting common brain region where all four groups have significant positive activation (P<0.05, FWE; with a minimum cluster size of 20 voxels). (DOCX) [file pone.0135468.s003.docx]

**Table S2.** Significant differences in brain activation during n-back task (2back>0back) between patients of schizophrenia and

young healthy controls with a common activation mask generated by interacting common brain region where all four groups

have significant positive activation (*P*<0.05, FWE; with a minimum cluster size of 20 voxels).

| Brain region | BA(L/R) | Left(L) | | |  |  | Right(R) | | |  |  |
| --- | --- | --- | --- | --- | --- | --- | --- | --- | --- | --- | --- |
|  |  | MNI coordinaes (in mm) | | | size | t-value  (voxels) | MNI coordinaes (in mm) | | | size | t-value  (voxels) |
|  |  | x | y | z |  |  | x | y | z |  |  |
| ***Patients > Controls*** |  |  |  |  |  |  |  |  |  |  |  |
| Putamen |  | -28 | 4 | 64 | 616 | 15.94 |  |  |  |  |  |
| Supplementary Motor Area | 6 | -14 | 2 | 68 |  | 9.65 |  |  |  |  |  |
|  | 40 | 38 | -36 | 40 | 292 | 11.27 |  |  |  |  |  |
| Inferior Parietal Lobule | 40 | -54 | -36 | 54 | 845 | 8.98 |  |  |  |  |  |
|  | 7 | -40 | -60 | 56 |  | 7.95 |  |  |  |  |  |
|  | 40 | -36 | -42 | 40 |  | 6.92 |  |  |  |  |  |
| Precuneus Gyrus | 7 | -12 | -66 | 46 | 296 | 8.97 |  |  |  |  |  |
| Supplementary Motor Area | 6 |  |  |  |  |  | 6 | 6 | 52 | 612 | 8.79 |
| Superior Frontal Gyrus | 8 |  |  |  |  |  | 22 | 6 | 54 |  | 8.22 |
| Supplementary Motor Area | 6 | -6 | 16 | 52 |  | 6.61 |  |  |  |  |  |
| Inferior Frontal Gyrus | 45 | -46 | 32 | 18 | 326 | 8.46 |  |  |  |  |  |
| Precentral Gyrus | 6 |  |  |  |  |  | 52 | 4 | 46 | 356 | 8.14 |
| Insula | 48 |  |  |  |  |  | 46 | 2 | 38 |  | 7.32 |
| Inferior Frontal Gyrus | 44 |  |  |  |  |  | 46 | 6 | 24 |  | 6.78 |
| Precentral Gyrus | 6 | -52 | 8 | 38 | 478 | 7.52 |  |  |  |  |  |
|  | 6 | -44 | -4 | 40 |  | 7.09 |  |  |  |  |  |
|  | 6 | -54 | 4 | 20 |  | 6.75 |  |  |  |  |  |
| Inferior Temporal Gyrus | 37 | -48 | -56 | -8 | 40 | 6.15 |  |  |  |  |  |
| Superior Parietal Lobule | 7 |  |  |  |  |  | 22 | -62 | 50 | 41 | 6.07 |
| Middle Frontal Gyrus | 46 |  |  |  |  |  | 36 | 54 | 18 | 28 | 5.32 |
|  | 45 |  |  |  |  |  | 50 | 44 | 18 | 28 | 5.25 |
|  | 46 |  |  |  |  |  | 38 | 38 | 24 |  | 4.78 |
| **Controls > Patients** |  |  |  |  |  |  |  |  |  |  |  |
| Middle Frontal Gyrus | 9 |  |  |  |  |  | 38 | 14 | 46 | 379 | 11.36 |
|  | 9 |  |  |  |  |  | 38 | 22 | 52 |  | 9.19 |
| Superior Frontal Gyrus | 8 |  |  |  |  |  | 24 | 24 | 62 |  | 6.22 |
| Precuneus Gyrus |  |  |  |  |  |  | 8 | -54 | 42 | 93 | 8.82 |
| Middle Frontal Gyrus | 9 | -38 | 12 | 58 | 64 | 7.70 |  |  |  |  |  |
| Angular Gyrus | 39 |  |  |  |  |  | 44 | -50 | 32 | 46 | 6.89 |
| Cerebelum |  | -14 | -74 | -28 | 59 | 6.00 |  |  |  |  |  |
