# Supplementary material for: Cerebral Inefficient Activation in Schizophrenia Patients and Their Unaffected Parents during the N-Back Working Memory Task: A Family fMRI Study
Source: PLoS One. 2015 Aug 13;10(8):e0135468. doi: 10.1371/journal.pone.0135468 (PMC4536207; doi:10.1371/journal.pone.0135468)
Supplement: S3 Table — Using a common activation mask generated by interacting common brain region where all four groups have significant positive activation (P<0.05, FWE; with a minimum cluster size of 20 voxels). (DOCX) [file pone.0135468.s004.docx]

**Table S3.** Significant differences in brain activation during n-back task (2back>0back) between unaffected parents of patients and

old healthy controls with a common activation mask generated by interacting common brain region where all four groups have

significant positive activation (*P*<0.05, FWE; with a minimum cluster size of 20 voxels).

| Brain region | BA(L/R) | Left(L) | | |  |  | Right(R) | | |  |  |
| --- | --- | --- | --- | --- | --- | --- | --- | --- | --- | --- | --- |
|  |  | MNI coordinaes (in mm) | | | size | t-value  (voxels) | MNI coordinaes (in mm) | | | size | t-value  (voxels) |
|  |  | x | y | z |  |  | x | y | z |  |  |
| ***Parents > Controls*** |  |  |  |  |  |  |  |  |  |  |  |
| Middle Frontal Gyrus | 45 |  |  |  |  |  | 36 | 44 | 14 | 675 | 11.50 |
| Superior Parietal Lobule | 7 | -22 | -68 | 58 | 265 | 8.37 |  |  |  |  |  |
| Inferior Parietal Lobule | 7 | -36 | -56 | 52 |  | 6.29 |  |  |  |  |  |
| Superior Parietal Lobule | 40 |  |  |  |  |  | 42 | -48 | 60 | 156 | 7.98 |
| Precuneus Gyrus | 7 |  |  |  |  |  | 14 | -72 | 48 | 173 | 7.86 |
| Middle Frontal Gyrus | 6 |  |  |  |  |  | 36 | -2 | 60 | 171 | 7.54 |
| Precentral Gyrus | 6 |  |  |  |  |  | 28 | -2 | 68 |  | 5.49 |
|  | 6 | -46 | 6 | 40 | 171 | 7.28 |  |  |  |  |  |
| Middle Frontal Gyrus | 6 | -36 | 2 | 52 |  | 5.16 |  |  |  |  |  |
|  | 6 | -30 | 2 | 44 |  | 5.06 |  |  |  |  |  |
| Precentral Gyrus | 44 |  |  |  |  |  | 54 | 10 | 36 | 83 | 6.53 |
| Middle Frontal Gyrus | 46 | -34 | 26 | 28 | 81 | 6.17 |  |  |  |  |  |
|  | 48 | -30 | 34 | 26 |  | 5.80 |  |  |  |  |  |
| Superior Frontal Gyrus | 6 | -20 | 2 | 66 | 59 | 5.99 |  |  |  |  |  |
|  | 8 |  |  |  |  |  | 28 | 30 | 52 | 73 | 5.78 |
| Middle Frontal Gyrus | 9 |  |  |  |  |  | 32 | 34 | 46 |  | 5.40 |
| Middle Occipital Gyrus | 39 |  |  |  |  |  | 40 | -64 | 26 | 23 | 5.35 |
| Supplementary Motor Area | 32 | -4 | 12 | 52 | 40 | 5.28 |  |  |  |  |  |
| ***Controls > Parents*** |  |  |  |  |  |  |  |  |  |  |  |
| Precuneus Gyrus |  | -8 | -56 | 42 | 119 | 7.93 |  |  |  |  |  |
| Middle Frontal Gyrus | 45 |  |  |  |  |  | 46 | 32 | 40 | 36 | 6.26 |
| Precuneus Gyrus |  |  |  |  |  |  | 12 | -56 | 44 | 44 | 6.23 |
| Inferior Frontal Gyrus | 47 | -40 | 24 | 6 | 25 | 5.99 |  |  |  |  |  |
| Cerebelum |  | -42 | -68 | -28 | 27 | 5.49 |  |  |  |  |  |
