# Supplementary material for: Cerebral Inefficient Activation in Schizophrenia Patients and Their Unaffected Parents during the N-Back Working Memory Task: A Family fMRI Study
Source: PLoS One. 2015 Aug 13;10(8):e0135468. doi: 10.1371/journal.pone.0135468 (PMC4536207; doi:10.1371/journal.pone.0135468)
Supplement: S4 Table — (DOCX) [file pone.0135468.s005.docx]

**Table S4. Cognitive (WCST and LM) performance in four groups: the patients, young healthy controls, parents and older unaffected controls.**

| **Variable** | **SCZ**  **(n=20)** | **NCS**  **(n=20)** | ***P*** ***^a^*** | **PAT**  **(n=20)** | **NCP**  **(n=20)** | ***P*** ***^a^*** |
| --- | --- | --- | --- | --- | --- | --- |
| **Executive control(WCST)** |  |  |  |  |  |  |
| Time | 8.75(4.55) | 8.00(5.01) | 0.62 | 10.35(3.28) | 10.60(5.76) | 0.87 |
| Trials Administered | 115.90(17.08) | 105.50(21.93) | 0.10 | 114.20(20.68) | 120.50(15.09) | 0.28 |
| Categories Completed | 3.90(2.27) | 4.75(2.02) | 0.22 | 3.15(2.41) | 2.85(2.32) | 0.69 |
| Total Correct Responses | 68.15(15.30) | 68.50(16.17) | 0.94 | 64.40(12.76) | 64.60(17.27) | 0.97 |
| Percent Correct Responses | 60.54(17.08) | 67.83(19.35) | 0.21 | 59.22(18.91) | 55.08(17.99) | 0.48 |
| Total Errors | 47.75(24.58) | 37.00(27.69) | 0.20 | 49.80(27.21) | 55.90(25.29) | 0.47 |
| Trials to Compete First Category | 36.75(37.18) | 28.40(31.08) | 0.45 | 34.25(35.87) | 44.95(42.52) | 0.40 |
| Percent Conceptual Level Responses | 47.59(22.28) | 59.07(24.55) | 0.13 | 44.62(25.66) | 39.00(23.31) | 0.47 |
| Perseverative Responses | 29.00(18.20) | 20.60(20.62) | 0.18 | 27.80(17.53) | 29.95(15.73) | 0.69 |
| Perseverative Errors | 25.10(14.24) | 18.15(15.98) | 0.16 | 24.35(14.22) | 26.65(13.12) | 0.60 |
| Percent Perseverative Errors | 20.78(10.23) | 15.78(11.62) | 0.16 | 19.92(10.13) | 21.46(9.51) | 0.62 |
| Nonperseverative Errors | 22.65(13.37) | 18.85(19.58) | 0.48 | 25.45(16.48) | 29.25(17.92) | 0.49 |
| Learning to Learn | 5.39(18.28) | 11.08(20.73) | 0.36 | 9.26(14.25) | 1.21(20.09) | 0.15 |
| **Episodic Memory(LM)** | 9.4(2.3) | 10.9(2.0) | 0.045 | 8.3(2.4) | 9.2(1.7) | 0.20 |

**Significance threshold defined at *p*＜0.05;P^a^ p value for Two-Sample *t* Test; WCST Wisconsin Card Sorting Test; LM Logical Memory from the Wechsler Memory Scale-Chinese Revised.**
